# IL-25 induces airways angiogenesis and expression of multiple angiogenic factors in a murine asthma model

**DOI:** 10.1186/s12931-015-0197-3

**Published:** 2015-03-18

**Authors:** Xiujuan Yao, Wei Wang, Yan Li, Ping Huang, Qian Zhang, Jingjing Wang, Wen Wang, Zhe Lv, Yunqing An, Jianguo Qin, Chris J Corrigan, Kewu Huang, Yongchang Sun, Sun Ying

**Affiliations:** Department of Respiratory Medicine, Beijing Tongren Hospital, Capital Medical University, Beijing, People’s Republic of China; Department of Immunology, School of Basic Medical Sciences, Capital Medical University, Beijing, People’s Republic of China; Department of Respiratory and Critical Care Medicine, Beijing Chao-Yang Hospital, Capital Medical University & Beijing Institute of Respiratory Medicine, Beijing, People’s Republic of China; Department of Laboratory Animal Sciences, Capital Medical University, Beijing, People’s Republic of China; Dongfang Hospital, The Second Clinical Medical College of Beijing University of Chinese Medicine, Beijing, People’s Republic of China; King’s College London, MRC & Asthma UK Centre in Allergic Mechanisms of Asthma, Division of Asthma, Allergy & Lung Biology, London, UK

**Keywords:** IL-25, IL-25 receptor, Asthma, Pathogenesis, Angiogenesis, Fibrosis, Murine model

## Abstract

**Background:**

Th2-promoting cytokine IL-25 might contribute to bronchial mucosal vascular remodelling in asthma through its receptor expressed by vascular endothelial and vascular smooth muscle cells.

**Methods:**

By utilising a newly established chronic asthma murine model induced by direct exposure of the airways to IL-25 alone, we examined effects of IL-25 on angiogenesis, vascular remodelling and expression of angiogenic factors, compared changes with those in a “classical” ovalbumin (OVA)-induced murine asthma model. IL-25 and OVA were intranasally instilled into the airways of BALB/c mice for up to 55 days. Airways vessels and angiogenic factors, including Von Willebrand Factor (vWF), amphiregulin, angiogenin, endothelin-1, transcription factor ERG, basic fibroblast growth factor (bFGF), epidermal growth factor (EGF), insulin-like growth factor (IGF-1) and vascular endothelial growth factor (VEGF) in lung sections, homogenates and BAL fluid were detected and quantified by immunostaining or enzyme linked immunosorbent assay (ELISA). An in house assay was also utilised to compare the effects of IL-25 and other Th2-cytokines on angiogenesis by human vascular endothelial cells.

**Results:**

Repetitive intranasal challenge with IL-25 alone or OVA alone in OVA-presensitised animals significantly increased peribronchial vWF ^+^ vessels in the murine airways, which was associated with remarkably elevated expression of amphiregulin, angiogenin, endothelin-1, bFGF, EGF, IGF-1, VEGF and ERG. IL-25, but not Th-2-cytokines induced human angiogenesis *in vitro*.

**Conclusions:**

The data suggest that chronic exposure of murine airways to IL-25 alone is able to reproduce a local angiogenic milieu. Thus, blocking IL-25 may attenuate vascular remodelling and improve outcomes in asthma patients.

## Introduction

Asthma is a chronic inflammatory disorder of the airways which is associated with airways hyperresponsiveness that leads to recurrent episodes of wheezing and breathlessness [1]. The pathophysiology of asthma has been intensively investigated. Nowadays, it is well accepted that in addition to inflammation, airways remodelling is also a hallmark feature of asthma which has been implicated in regulating disease severity and control [2-4]. Airways remodelling refers to structural changes including loss of epithelial integrity, thickening of the subepithelial basement membrane caused by fibrosis, goblet cell metaplasia and submucosal gland enlargement, increased airway smooth muscle mass, increased airways vascularity and vascular remodelling [2-4]. Although the notion that airways remodelling is one of the key features of asthma is well established, the underlying mechanisms have not been fully clarified. It is now hypothesised that inflammation and vascular remodelling in asthma are partially interdependent, reflecting a dysregulated wound healing process resulting in extracellular matrix deposition and angiogenesis [5].

Angiogenesis of bronchial mucosal vessels may be observed in response to a wide variety of stimuli, including chronic airways inflammation. It may be defined as either the formation of new vessels by sprouting from pre-existing vessels and/or lengthening and enlargement of existing vessels. Angiogenesis is a complex multiphase process, potentially involving a great number of growth factors, cytokines, chemokines, enzymes and other factors [4,6]. These are produced by resident stromal or inflammatory cells, creating a “vascular remodelling milieu”. Notwithstanding that vascular endothelial growth factor (VEGF) is considered to be a key mediator in this process, the specific roles of other molecules remain to be defined [6].

IL-25 (IL-17E), a distant member of the IL-17 family has been demonstrated to induce Th2-type immune responses, indicating that it may contribute to the pathogenesis of asthma and thereby this “vascular remodelling milieu” [7]. In mice, overexpression or exogenous administration of IL-25 produces an asthma-like inflammatory response, including increased serum IgE production, blood eosinophilia and lung eosinophilic infiltrates, epithelial cell hyperplasia/hypertrophy, increased mucus secretion and airways hyperreactivity [8-11]. Conversely, blockade of IL-25 remarkably reduced Th2 cytokine production and airways inflammation [7,12], and in addition ameliorated airways remodelling changes including peribronchial collagen deposition and smooth muscle hyperplasia, with corresponding reduction of hyperreactivity [12].

Our recent previous data further underline the ability of IL-25 to effect airways inflammation and fibrosis *in vivo*. Firstly, we detected elevated expression of IL-25 and its receptor in the human asthmatic bronchial mucosa at baseline and following allergen challenge [13]. Secondly, we showed that chronic intranasal instillation of IL-25 into murine airways is able to induce acute and chronic inflammatory responses reminiscent of human asthma [14]. Thirdly, we showed that IL-25, by binding to its IL-25R/IL-17RB, but not its IL-17RA receptor enhanced human lung vascular endothelial cell proliferation and angiogenesis *in vitro* by increasing the expression by these cells of basic fibroblast growth factor (bFGF) and VEGF and VEGF receptors [15,16]. Nevertheless, there is still a paucity of information as to the full range of potential effects of IL-25 in promoting a “vascular remodelling milieu” in the setting of asthmatic inflammation *in vivo*. In particular, angiogenic and growth factors such as epidermal growth factor (EGF), ETS-related gene (ERG), insulin-like growth factor 1 (IGF-1), amphiregulin, angiogenin and endothelin-1, together with transforming growth factor-β1 (TGF-β1), VEGF and bFGF have been suggested to have potential to participate in this “remodelling milieu”[4].

Consequently we set out to extend our previous studies to examine the direct effects of IL-25 alone on the production of these mediators in the airways in vivo in our murine model of chronic asthma. In this model, we compared in parallel the effects of direct intranasal instillation of IL-25 with those of intranasal OVA administered to mice pre-sensitised to mount an IgE response to OVA by intraperitoneal sensitization (a “classical” murine asthma model). Finally, we compared the direct effects of IL-25 with those of IL-4, IL-5 and IL-13 in promoting angiogenesis in a human *in vitro* model.

## Methods

### Animals

Female BALB/c mice (8–10 wk old) were purchased from Vital River Laboratories (Beijing, China) and maintained in a pathogen free mouse facility located in the Department of Laboratory Animal Sciences, Capital Medical University, Beijing, China. They were kept in 12 h light and 12 h dark with free access to food and water. All experiments were approved by the Institutional Animal Care and Use Committee (IACUC).

### Allergen- and IL-25 intranasal instillation protocol

Mice were divided into 3 groups, including a group serially challenged with OVA (a “classical” allergen-induced asthma model) [14,17], a group challenged with saline as a negative control and a group serially challenged with IL-25. Briefly, OVA-challenged group were first sensitized by intraperitoneal injection of OVA (Sigma-Aldrich, Beijing, China, 100 μg emulsionised in Al[OH]3/dose) on days 0 and 12. Then 50 μg of OVA (OVA50) in 50 μL saline/dose were further administered to these mice daily by nasal instillation from days 18 to 23. IL-25 challenged group were not sensitized to OVA on days 0 and 12, but suffered daily nasal instillation with recombinant mouse IL-25 (mIL-25, R&D Systems, 2 μg in 50 uL saline) from days 18 to 23 [14,18]. Subsequently, these mice were further challenged intranasally either with OVA (OVA50 group) or with IL-25 (IL-25 group) every 2 days for a further 30 days. 5 mice in each group were observed for a further 17 days after stopping the challenges. Saline negative group was intraperitoneally injected with the same amount of Al[OH]3 on days 0 and 12, then nasally administered with saline at time points corresponding to those in the other groups.

### Bronchoalveolar lavage fluid collection and preparation of lung tissue homogenates

Bronchoalveolar lavage fluid (BALF) was collected from mice immediately following euthanasia as previously described [14]. Supernatants were stored at - 80°C until used. Right lung tissue (100 mg) was homogenized in 2 mL PBS containing 1% Triton-X100 and a protease inhibitor cocktail tablet (Roche Diagnostics GmbH, Mannheim, Germany). Debris was removed by centrifugation and the supernatants collected and stored at - 80°C until used for measurement of analytes.

### Analysis of VEGF and bFGF

VEGF and bFGF concentrations in BALF and lung homogenates were measured using commercial ELISA kits (eBioscience, San Diego, CA). In brief, 100 μl of capture antibody were added to each plate well overnight at 4°C. After washing 5 times, 100 μl of acidified BALF or lung homogenate were added to the plate wells which were then incubated for 2 h at room temperature (RT). After 5 washes, 100 μl of detection antibody were added to each well and the plates incubated for a further 1 hr at 37°C. After washing, 100 μl of avidin-HRP labelled antibody were added and the plates incubated for 30 min at RT. Plates were washed again 5 times and 100 μl of substrate solution added to each well for incubation at RT in the dark. After colour development, 50 μl of stop solution were added to each well. Absorbance was measured at 450 nm using a GloMAc-Multi Detection System (e7801, Shanghai Promega Biological Products, Ltd, China). Concentrations of VEGF and bFGF in BALF and lung homogenates were determined by interpolation from the standard curve.

### Lung immunohistochemistry

Immunohistochemistry was used to detect bronchial vascular biomarkers. The primary monoclonal antibodies against mouse transcription factor (ERG, 1:80), epidermal growth factor (EGF, 1:100), insulin-like growth factor (IGF-1, 1:400), endothelin-1 (1:600), angiogenin (1:400) and amphiregulin (1:500) were purchased from Abcam (Hong Kong, China). The primary monoclonal antibody against mouse Von Willebrand factor (vWF, 1:50) was purchased from CHEMICON International, Inc. MA 01730. The PAP (peroxidase anti-peroxidase) technique was used as previously described [13-15]. Positively staining cells were detected using the glucose oxidase-DAB-nickel method [19]. At least 8 random high-power fields (200 × total magnification) of each mice in the 3 groups should be independently analysed by 2 observers in a blinded fashion on a Leica DM6000B microscope (Leica, Wetzlar, Germany) connected with a Leica Application Suite Version 3.6. The data were expressed as percentages of positive stained areas per unit area of entire lung sections.

### In vitro angiogenesis assay

It is believed that Th2-type cytokines such as IL-4, IL-5 and IL-13 play a significant role in airways remodelling in asthma. To further investigate whether these cytokines also have effects on angiogenesis, we used a well established in vitro angiogenesis assay (AngioKit; TCS CellWorks, Buckingham, UK) [15,16], which is based on co-culture of HUVEC over a monolayer of irradiated fibroblasts. Briefly, cultures were incubated at 37°C in a humidified atmosphere with 5% CO_2_. Culture medium with recombinant human IL-4, IL-5, IL-13 and IL-25 (10 ng/ml, R&D Systems, Abingdon, UK) [15,16] was replenished on days 4, 7, and 9. Culture medium alone and recombinant human VEGF-A (R&D Systems, 10 ng/ml) [15,16] served as negative and positive assay controls, respectively. On day 11 cultured cells were fixed and vascular structures visualized by labelling with mouse anti-CD31 according to the manufacturer’s instructions. Multiple photomicrographs (×4 objective) (4 separate fields per well) were taken at clock points 12, 3, 6 and 9, and angiogenesis in each field of view quantified using image analysis software (AngioSys; TCS CellWorks) as described previously [15,16]. The analysis software segmented the images using a grey level threshold tool to select CD31-labelled cells. The resultant binary images were skeletonized and branch points removed to determine the total lengths of individual tubules. Branch points were counted and the total area of CD31 labelling determined from the original binary images, permitting overall numbers of vascular junctions, tubules, and tubule length to be determined.

### Statistical analysis

The results were analyzed using InStat 2.01 software (GraphPad, San Diego, CA, USA). Differences between experimental groups were analyzed using 2-way parametric ANOVA followed by a Bonferroni post test or the Student’s unpaired *t* test. Data are presented as the mean ± SEM. For all tests, p values less than 0.05 were considered significant.

## Results

### IL-25 increased airways vascularity

**Figure 1:**
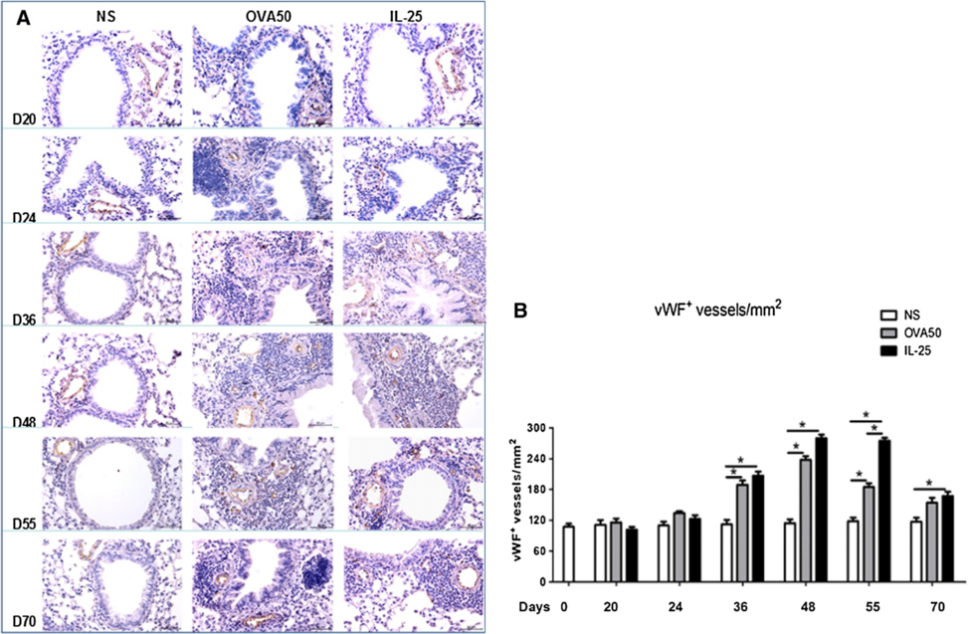
**IL-25 increased airways vWF expression. A**: Representative photomicrographs of von Willebrand factor (vWF) immunoreactivity in lung sections from saline (NS)-, OVA- and IL-25-challenged mice at various time points as indicated (original magnification x20). **B**: Quantitative analysis of vWF^+^ blood vessels per unit area of lung sections. Data were collected from 3 independent experiments and are expressed as the mean ± SEM (n = 5 in each group of mice at each time point).*p < 0.05.

**Figure 2:**
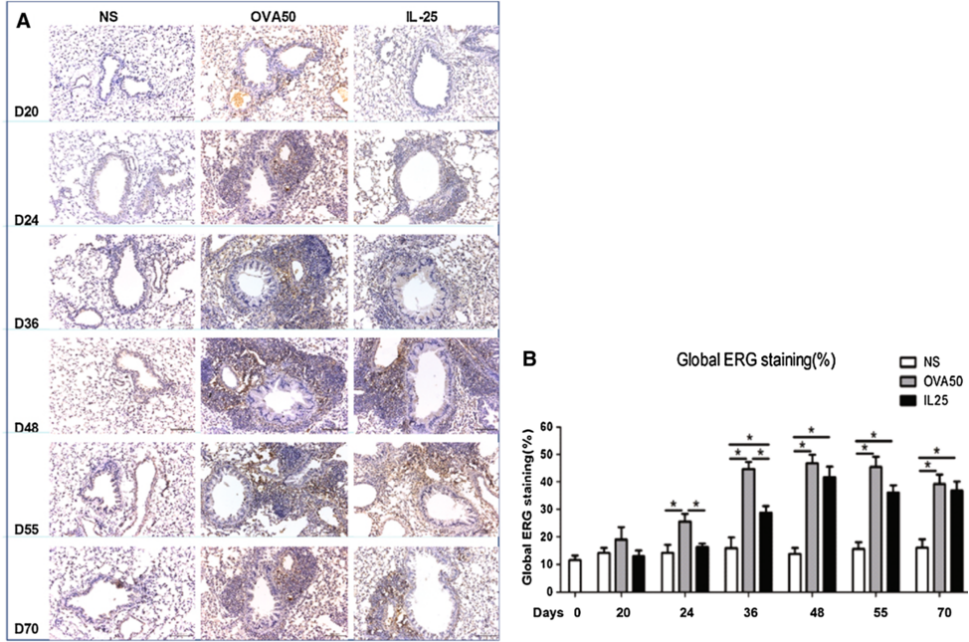
**IL-25 increased airways ERG expression. A**: Representative photomicrographs of ETS-related gene (ERG) immunoreactivity in lung sections from saline (NS)-, OVA- and IL-25-challenged mice at various time points as indicated (original magnification x20). **B**: Quantitative analysis of ERG immunoreactivity. The data were collected from 3 independent experiments and are expressed as the mean ± SEM (n = 5 in each group at each time point). *p < 0.05.

Immunoanalysis showed that IL-25, as compared with saline challenge induced marked airways angiogenesis as indicated by significantly elevated numbers of peri-bronchial, vWF^+^ immunoreactive blood vessels from day 36 and persisting until the end of the experiment (day 70) (Figure 1). Similarly, OVA challenged animals also showed elevated vWF^+^ immunoreactive vessels from days 36 to 55 which however declined by day 70 (Figure 1). Correspondingly, IL-25 challenge also significantly increased the expression of global airways ERG immununoreactivity from day 36 until day 70 (Figure 2). OVA challenge similarly increased ERG expression although this was evident slightly earlier from day 24 until the end of the experiment (Figure 2).

### IL-25 potentiated airways parenchymal and luminal VEGF and bFGF expression

**Figure 3:**
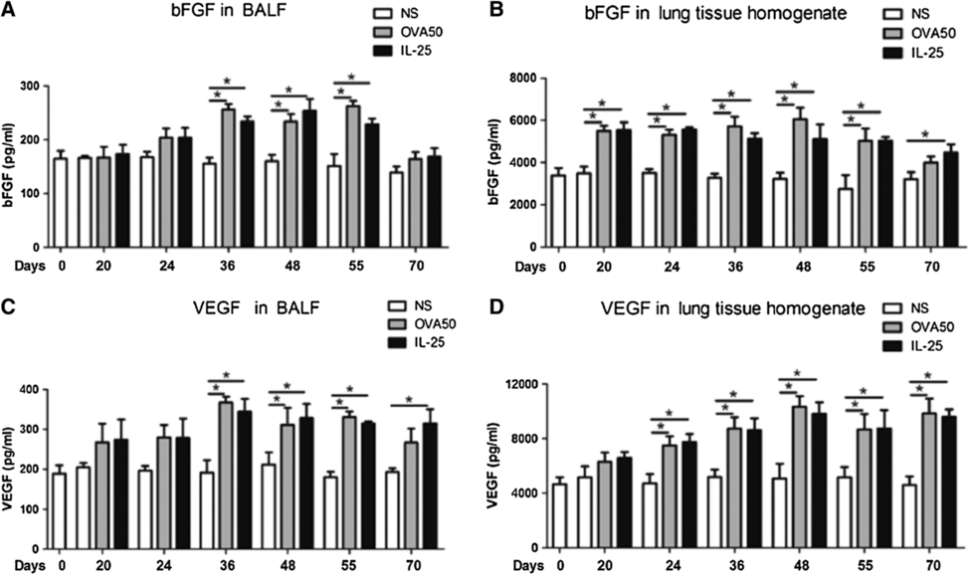
**IL-25 potentiated airways VEGF and bFGF expression.** Concentrations of basic fibroblast growth factor (bFGF, **A** and **B**) and vascular endothelial growth factor (VEGF, **C** and **D**) in bronchoalveolar lavage fluid (BALF) and lung tissue homogenates from saline (NS)-, OVA- and IL-25-challenged mice at various time points as indicated. The data were collected from 3 independent experiments and are expressed as the mean ± SEM (n = 5 in each group at each time point). *p < 0.05.

IL-25, as compared with saline challenge of the murine airways induced significantly elevated mean concentrations of bFGF, as measured by ELISA, in lung homogenates from day 20 and in BALF from day 36, with resolution in BALF by day 70 (Figure 3). Mean VEGF concentrations were also significantly elevated in lung homogenates from day 24 and BALF from day 36 until the end of the experiment. OVA challenge induced similar changes in bFGF and VEGF concentrations in both compartments with a similar time course (Figure 3).

### IL-25 induced airways EGF and IGF-1 expression

**Figure 4:**
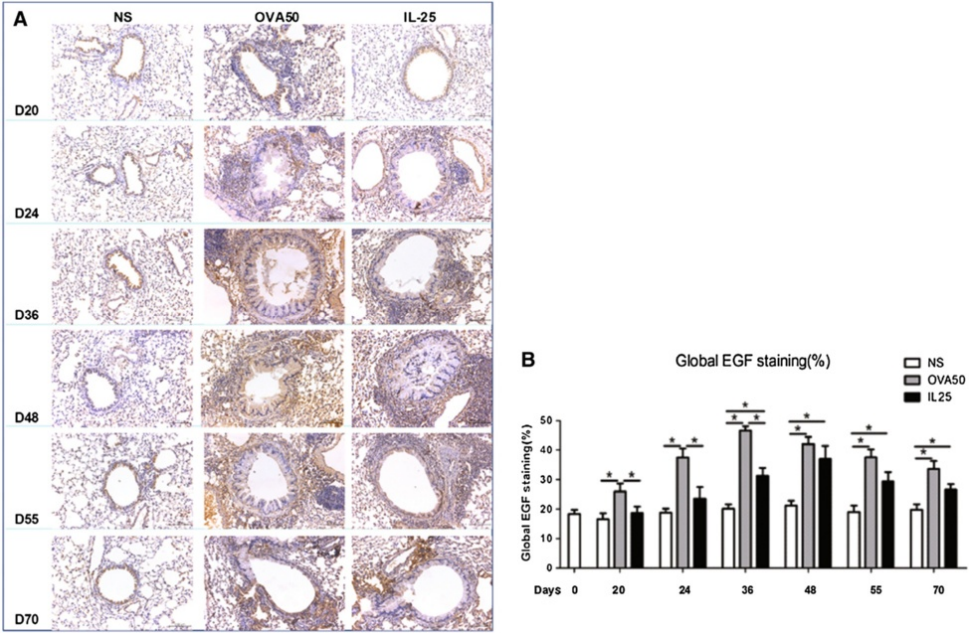
**IL-25 induced airways EGF expression. A**: Representative photomicrographs of epidermal growth factor (EGF) immunoreactivity in lung sections from saline (NS)-, OVA- and IL-25-challenged mice at various time points as indicated (original magnification x20). **B**: Quantitative analysis of EGF immunoreactivity. The data were collected from 3 independent experiments and are expressed as the mean ± SEM (n = 5 in each group at each time point). *p < 0.05.

**Figure 5:**
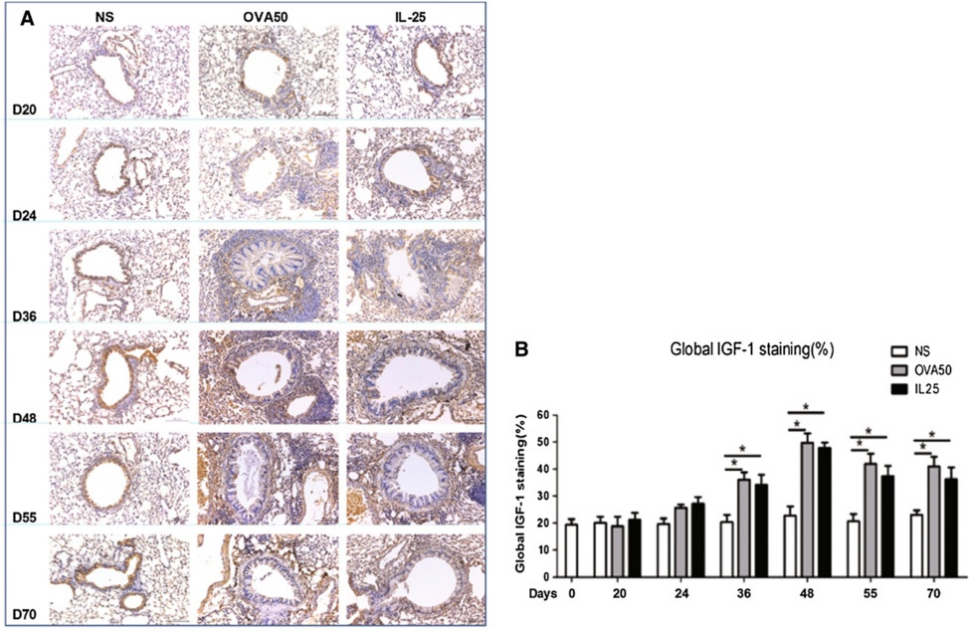
**IL-25 induced airways IGF-1 expression. A**: Representative photomicrographs of insulin-like growth factor 1 (IGF-1) immunoreactivity in lung sections from saline (NS)-, OVA- and IL-25-challenged mice at various time points as indicated (original magnification x20). **B**: Quantitative analysis of IGF-1 immunoreactivity. The data were collected from 3 independent experiments and are expressed as the mean ± SEM (n = 5 in each group at each time point). *p < 0.05.

Immunoanalysis showed that IL-25, as compared with saline challenge of the airways significantly increased the expression of lung and peribronchial EGF and IGF-1 immunoreactivity from day 36 until the end of the experiment (Figures 4 and 5). OVA challenge produced a similar effect which was apparent earlier (from day 20) and more marked between days 20 and 36 in the case of EGF (Figures 4 and 5).

### IL-25 induced elevated airways endothelin-1, angiogenin and amphiregulin expression

**Figure 6:**
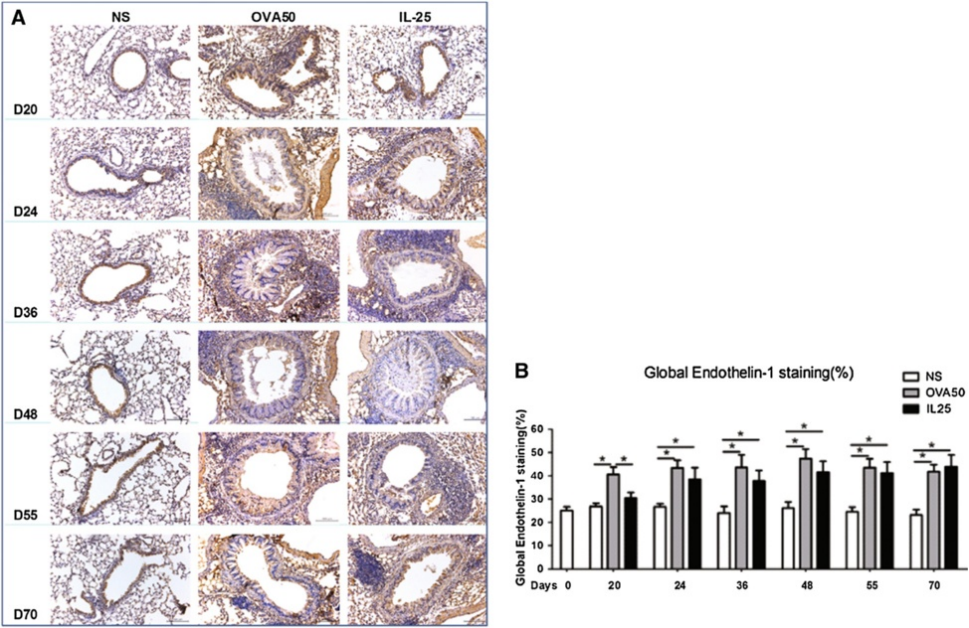
**IL-25 induced airways endothelin-1 expression. A**: Representative photomicrographs of endothelin-1 immunoreactivity in lung sections from saline (NS)-, OVA- and IL-25-challenged mice at various time points as indicated (original magnification x20). **B**: Quantitative analysis of endothelin-1 immunoreactivity. The data were collected from 3 independent experiments and are expressed as the mean ± SEM (n = 5 in each group at each time point). *p < 0.05.

**Figure 7:**
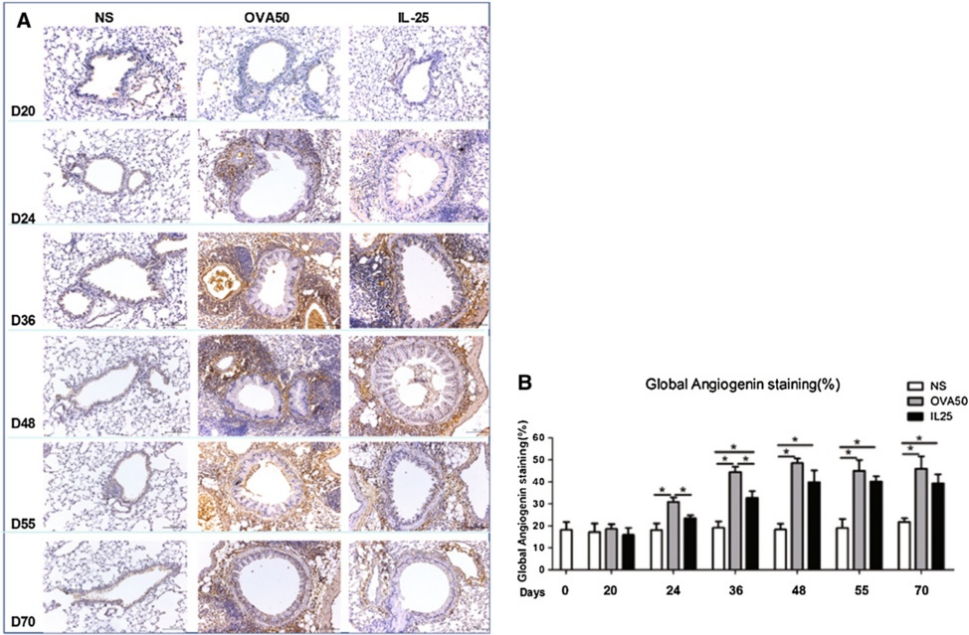
**IL-25 induced airways angiogenin expression. A**: Representative photomicrographs of angiogenin immunoreactivity in lung sections from saline (NS)-, OVA- and IL-25-challenged mice at various time points as indicated (original magnification x20). **B**: Quantitative analysis of angiogenin immunoreactivity. The data were collected from 3 independent experiments and are expressed as the mean ± SEM (n = 5 in each group at each time point). *p < 0.05.

**Figure 8:**
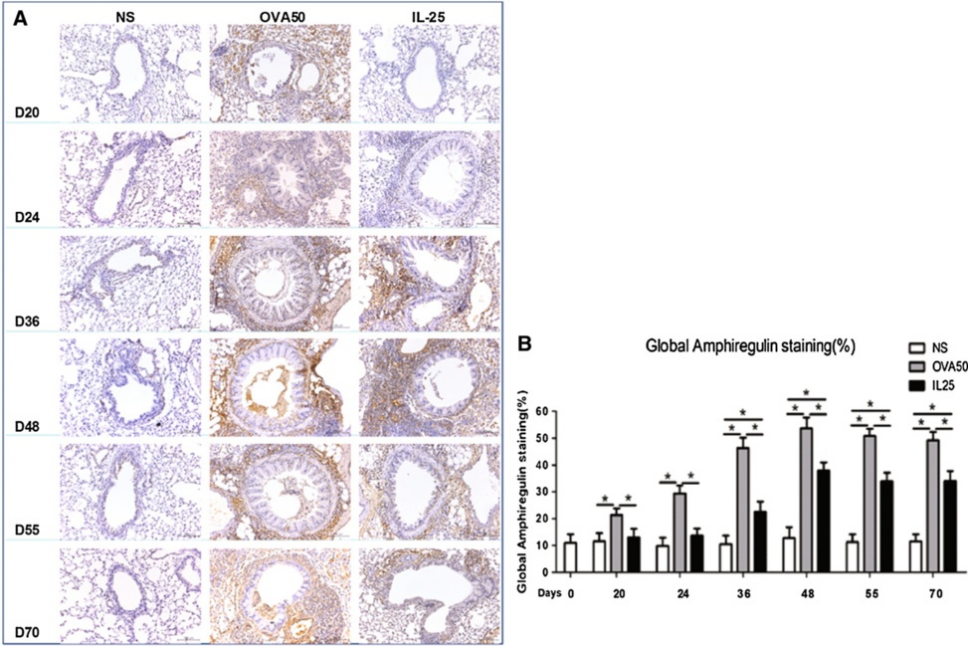
**IL-25 induced airways amphiregulin expression. A**: Representative photomicrographs of amphiregulin immunoreactivity in lung sections from saline (NS)-, OVA- and IL-25-challenged mice at various time points as indicated (original magnification x20). **B**: Quantitative analysis of amphiregulin immunoreactivity. The data were collected from 3 independent experiments and are expressed as the mean ± SEM (n = 5 in each group at each time point). *p < 0.05.

Immunoanalysis showed that IL-25 as compared with saline challenge of the airways significantly increased expression of lung and peribronchial endothelin-1, angiogenin and amphiregulin immunoreactivity from day 24 (endothelin-1) or day 36 until the end of the experiment. OVA challenge induced similar but more marked changes which were apparent earlier (From days 20 or 24) (Figures 6, 7 and 8).

### IL-25, but not IL-4, IL-5 or IL-13 induced angiogenesis in vitro

**Figure 9:**
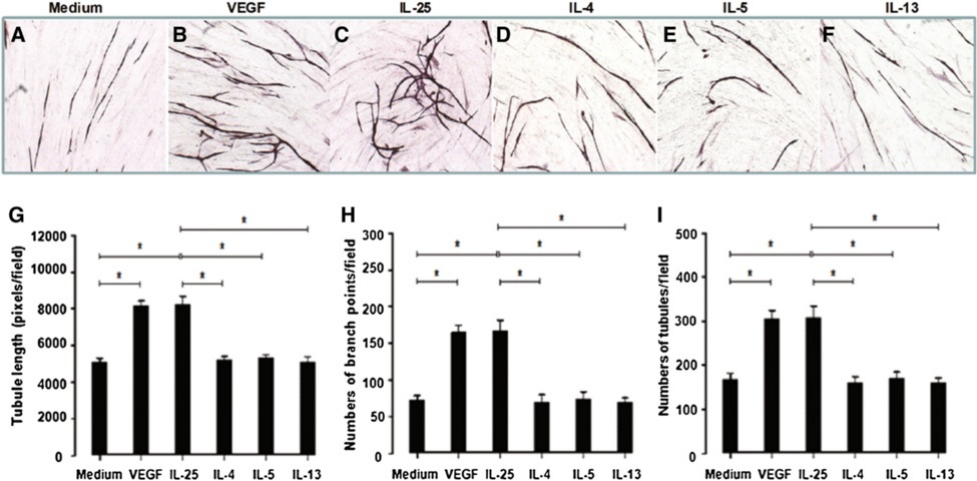
**IL-25, but not IL-4, IL-5 or IL-13 induced angiogenesis in vitro.** Top panel: representative light photomicrographs (4x original magnification) show formation of primitive vascular tubule structures by human vascular endothelial cells after 11 days of culture (top panel) with medium **(A)**, VEGF (10 ng/mL) **(B)**, IL-25 (10 ng/mL) **(C)**, IL-4 (10 ng/mL) **(D)**, IL-5 (10 ng/mL) **(E)** and IL-13 (10 ng/mL) **(F)**. Bottom panel: computer-assisted quantification of total tubule lengths **(G)**, numbers of branch points **(H)** and total numbers of tubules **(I)**. Bars show the mean ± SEM of three separate experiments performed in duplicate. *p < 0.05. Mann–Whitney *U* test. *p < 0.05.

In an *in vitro* angiogenesis assay, IL-25, but not IL-4, IL-5 or IL-13, induced lengthening and branching of microvascular tubules by HUVEC as compared with VEGF, which was used as a positive control (Figure 9).

## Discussion

Accumulating reports have firmly established angiogenesis and vascular remodelling as features of asthma pathophysiology which could have an important impact on day to day symptomatology as well as the natural history of the disease [20-22]. So far, relatively little is known about the mechanisms of these changes. Here we demonstrate that chronic challenge of murine airways with IL-25 alone has the capacity to induce angiogenesis *in vivo* and increase local production of a variety of angiogenic mediators in a setting of chronic inflammation resembling that induced by “classical” murine asthma models which require prior IgE sensitization of the animals. The only difference was that some of the changes were more marked and some occurred slightly earlier with OVA, as compared with IL-25 challenge, perhaps at least partly reflecting the release of mast cell mediators in the airways. This does not of course imply that the molecular mechanisms inducing these changes are necessarily similar, but does underline the potential pleiotropic activities of IL-25 alone. Further, we have shown that, although a variety of Th2 cytokines as well as IL-25 have been implicated in causing remodelling changes in human and murine airways in asthma, only IL-25, and not IL-4, IL-5 or IL-13 is able directly to induce angiogenesis by human vascular endothelial cells. This extends our previous data [16] and is also congruent with previous study [12] showing that angiogenesis is increased in the airways of mice sensitized to, then challenged with house dust mite and that this can be abrogated by IL-25 blockade, possibly at least partly by reduced recruitment of CXCR2 endothelial cell progenitors from the bone marrow.

Vascular endothelial growth factor (VEGF) is currently considered to be a key angiogenic factor in asthma [23], while the expression of bFGF is also upregulated in human asthma and has been correlated with the increased vascularity of the bronchial mucosa and lung function [24,25]. Our data showed that both IL-25 and OVA-challenged mice showed increased production of both mediators, first in the lung parenchyma and in the airways lumen, with a time course reflecting that of elevated blood vessel signature markers including vWF and ERG. vWF is a common marker for evaluating angiogenesis *in vivo* and *ex vivo* [12], while ERG is a member of the ETS (erythroblast transformation-specific) family of transcription factors which is selectively expressed by endothelial cells, specific haematopoetic cells and pre-cartilage cells. It has been shown that ERG plays an important role in blood vessel homeostasis and angiogenesis by regulating a variety of endothelial cellular functions including survival, junctional stability and migration [26].

Physiologically, airways blood vessel homeostasis likely reflects a balance between the production of endogenous angiogenic activators and inhibitors. When the balance is perturbed because of over-production of angiogenic factors and/or down-regulation of inhibitors, a pro-angiogenic niche ensues and aberrant vascularisation occurs [2-4]. Aside from VEGF and FGF, which are heparin binding growth factors, several other pro-angiogenic mediators have been implicated in regulating neovascularisation in airways. Circulating concentrations of insulin-like growth factor (IGF-I) have been linked with airways vascular remodelling in patients with chronic airways disease [27]. Inhibition of epidermal growth factor (EGF) receptor signalling ameliorated airways hyperreactivity and remodelling in a murine model of chronic asthma [28]. Amphiregulin, a member of the EGF family and another ligand of the EGF receptor, has been shown to promote proliferation of airways epithelial and smooth muscle cells and mucin gene expression [29]. Neutralization of amphiregulin also prevented cultured endothelial cells from forming tubes [30]. In addition to growth factors, other pro-angiogenic mediators such as endothelin-1 and angiogenin also contribute to aberrant intrapulmonary neovascularisation and remodelling [31-33]. Endothelin-1, in addition to promoting synthesis of ECM proteins by epithelial cells and fibroblasts, myofibroblast differentiation and proliferation of mesenchymal cells, also induces migration and proliferation of vascular cells [34]. Angiogenin, first isolated from conditioned medium of colonic carcinoma cell cultures, is a potent tumour-derived angiogenic factor but also plays a role in several non-malignant vasculoproliferative disorders, and like VEGF induces vascular endothelial cell proliferation, migration and tubule formation [35]. Our data show that all of these pro-angiogenic agents have the potential to be upregulated by IL-25 alone in the setting of asthma-like inflammation of the airways *in vivo*, with a time course congruent with that of angiogenesis.

## Conclusions

Our data do not distinguish between direct and indirect effects of IL-25 in causing angiogenesis, although we have previously shown [15,16] that IL-25 acts directly on endothelial cells to induce angiogenesis, albeit at least partly through increasing VEGF and bFGF expression by these cells, whereas other Th2-type cytokines (IL-4, IL-5, IL-13), as shown in this study, do not. Angiogenesis likely involves many cell types including fibroblasts and vascular smooth muscle cells [2-6]. We have further preliminary data suggesting that IL-25 also induces expression of VEGF and EGF fibrotic factors by human lung fibroblasts (in preparation). We conclude that IL-25 may be a master switch for the production of many important pro-angiogenic mediators by a diverse variety of airways structural cells. Targeting IL-25 may provide a novel therapeutic approach to altering the natural history of asthmatic inflammation and associated symptoms.

## Abbreviations

AHR: Airways hyperresponsiveness
bFGF: Basic fibroblast growth factor
ECM: Extracellular matrix
EGF: Epidermal growth factor
ERG: ETS-related gene ETS, erythroblast transformation- specific
HUVEC: Human umbilical vein endothelial cells
IGF-1: Insulin-like growth factor 1
IHC: Immunohistochemistry
IL-25: Interleukin 25
IL-25R: Interleukin-25 receptor
OVA: Ovalbumin
VEGF: Vascular endothelial growth factor
vWF: Von Willebrand factor
